# Promoting Safe Sleep: The Role of Doulas in Black Communities

**DOI:** 10.1007/s10995-024-03928-0

**Published:** 2024-06-04

**Authors:** Stephenie Howard, Shannon Dancy

**Affiliations:** School of Social Work, Norfolk State University, Brown Memorial Hall, 700 Park Ave, Norfolk, VA 23504 USA

**Keywords:** Doulas, Alternative care, Sudden infant death syndrome (SIDS), Infant death, Safe sleep

## Abstract

**Background:**

Accidental suffocation and strangulation in bed continues to be a critical issue in Black communities, despite the widespread initiatives to promote safe sleep. Doulas are in an ideal position to promote safe sleep, particularly in hard-to-reach communities that are more distrusting of conventional medical providers. Little is known about their practices and perspectives for putting infants down to rest. This study informs this gap in the literature.

**Purpose:**

The purpose of this study was to explore doulas’ perspectives and practices in the field of putting infants down to sleep. The researchers aimed to determine whether Black caregivers that work with doulas are likely to encounter safe sleep education.

**Methods:**

The researchers used a descriptive approach to inquiry. They conducted three focus groups with a total of 17 Black doulas. The researchers independently and critically reviewed the transcriptions and observation notes from each group to identify codes. They then triangulated the results using Artificial Intelligence-driven tools.

**Findings:**

The study found four themes: (1) Individualized Services, (2) Cultural Sensitivity, (3) Negotiating Safety, and (4) Safe Sleep Education.

**Conclusions:**

The study concluded doulas have a commitment to promoting safe sleep. The researchers found that doulas engage in practices that help caregivers to integrate safe sleep practices into their lifestyle and to adapt them to meet their needs. The researchers also documented a desire for more information and instruction on safe sleep among practicing doulas.

**Supplementary Information:**

The online version contains supplementary material available at 10.1007/s10995-024-03928-0.

## Significance

Scholars recognize that doulas play a vital role in infant and maternal health in Black communities, but little is known about their role in promoting safe sleep. This study contributes to this knowledge base, revealing that doulas uphold a commitment to safe sleep practices and are integral in helping Black caregivers negotiate safe sleep.

**Supplementary Information:**

The online version contains supplementary material available at 10.1007/s10995-024-03928-0.

This paper reports on the results of a qualitative study about the perspectives and practices of doulas in putting infants down for sleep. It helps to fill a critical gap in the literature. The findings have important implications for practices and policies for doulas. Given recent initiatives to include doula services in Medicaid coverage in Virginia, this paper places emphasis on the state of Virginia.

To frame the problem and its context a background section follows this brief introduction. Next, the authors synthesize the literature and identify the gaps in the existing knowledge base. Preceding the methodology section, the authors explain the purpose and objectives of this study. The authors then elaborate on the results of this study, followed by a discussion on the implications of policies and practices.

## Background Information

The CDC (2022b) documents that the infant mortality rate in 2020 in the US was 5.4 deaths per 1,000 live births. Allen and colleagues (2021) point to a critical developmental period in which infants are most vulnerable to sudden unexplained infant death (SUID). The age distribution of SUID is relatively low in the first month of life and peaks at 2–3 months. Approximately 90% of deaths occur before the age of six months (Allen et al., 2021).

Most infant deaths occur while the infant is asleep or in a sleep environment (i.e., crib, bed, etc.; Herman et al., 2015). Nationally, in 2020, there were 1,389 deaths due to sudden infant death syndrome (SIDS), 1,062 deaths due to unknown causes, and 905 deaths due to accidental suffocation and strangulation in bed (ASSB; CDC, 2022a). ASSB refers to incidents where a child unintentionally suffocates or becomes strangled while sleeping or resting in a bed or other sleeping environment. The American Academy of Pediatrics (AAP) introduced the term “Accidental Suffocation and Strangulation in Bed” to distinguish these incidents from SIDS, which is defined as the sudden death of an infant that cannot be explained after a thorough investigation. Researchers believe that most, if not all, ASSB cases are preventable.

To prevent ASSB infant fatalities, AAP developed safe sleep recommendations in 1992 and initiated the Back to Sleep (now known as Safe to Sleep) campaign in 1994 (CDC, 2022b). The AAP Task Force on SIDS recently updated its recommendations to prevent all sleep-related infant deaths in 2022 (Moon et al., 2022). They recommend placing the infant supine for sleep, having the infant sleep in the same room as the parent but on a separate surface (e.g., crib, bassinet, playpen), and removing all soft bedding and bumper pads from the infant’s sleep area. Readers should note that this paper will herein refer to these recommendations as “safe sleep.”

Despite the widespread efforts to promote safe sleep, CDC (2022b) research indicates that ASSB among infants has continued to rise since 1994. In 2020, the rate was 25.0 deaths per 100,000 live births (CDC, 2022b). The Virginia State Child Fatality Review Team (2018) determined the top four risk factors for sleep-related deaths often appear concurrently: soft bedding, such as blankets, pillows, and stuffed animals (93%); sleeping on an inappropriate sleep surface, such as a couch (85%); co-sleeping/bed-sharing (52%); and second-hand smoke (50%). Observations from the Eastern Virginia Child Fatality Review Team case records suggests that a major risk factor for ASSB is when the family experiences changes or disruptions such as caregiver illness, unplanned visitors, or unexpected family events . They also found the infant being fussy or sick was to be a risk factor.

It should further be noted that researchers have identified age-related risk factors for infant death. For example, Allen and colleagues (2021) maintain that death associated with bed-sharing is higher in younger infants, particularly those under 3 months of age, while death in older infants is more associated with them changing their sleep position from side or back to prone. Further exploring age-related risk factors, they found two broad categories: (1) Smoking during pregnancy and factors linked to disadvantaged racial categories and lower socio-economic status were associated with a younger age of death and (2) Factors linked to pregnancy and delivery complications, as well as extremes in birthweight and gestation duration, are initially associated with an older chronological age of death. However, after accounting for gestation, these factors were actually associated with a younger corrected age. This observation supports the notion that premature birth delays the vulnerable developmental window to a later postnatal age, as the infant was born at an earlier gestational age. Taken as a whole, the research supports the need for tailored interventions addressing both prenatal and postnatal factors.

### ASSB in Black Communities

Black infants are at exceptionally high risk for sudden unexplained infant deaths. They are twice as likely as White infants to die from SUIDs (CDC, 2022b). Moreover, Black infants have the second highest fatality rate for ASSB after Non-Hispanic American Indian/Alaska Native infants (CDC, 2022a). The Virginia Department of Social Services unfortunately reports the rates of ASSB in Black communities have been on the rise since 2019 (C. Lansden, personal communication, December 5, 2022). According to the Virginia Department of Social Services, these cases have primarily involved unsafe sleeping practices in conjunction with exposure to second-hand smoke, including THC (C. Lansden, personal communication, December 5, 2022).

Past studies explain reasons why infant death disproportionately affects Black communities. Herman et al. (2015) observed that distrust in conventional helping professionals influence whether caregivers will implement safe sleep recommendations. Stiffler et al. (2018) add that Black mothers believed infant death occurs randomly or at God’s will. They point to tradition and generational wisdom as more influential than professional judgment or textbooks among Black mothers. They attributed resistance to a cultural distrusting of outsiders, particularly from the medical field. Overall, the research points to a lack of education from trusted sources about the dangers of unsafe sleep.

Existing literature points to racial differences in adoption of the safe sleep practices recommended by the AAP. In a previous study using data from the National Infant Sleep Position (NISP) study, researchers found that Black infants bed-share more than other ethnic groups (Colson et al., 2013). They also observed a progressive increase in usual bed-sharing among Black infants over time. Specifically, for Black infants, the percentage of usual bed-sharing rose from 24.5% in 1993 to 39.6% in 2010, representing a significant increase (Colson et al., 2013. These findings are consistent with the rising rates of ASSB in Black communities, suggesting a need for behavioral interventions to support safe sleep in Black communities.

### Doula Services

Some new and expectant mothers supplement or replace the services of healthcare professionals with those provided by doulas. The Virginia Department of Health website (n.d.) describes doulas as professionals who “educate mothers to be healthy and have healthy babies and empower them to confidently make some of the most important decisions of their lives.” They work with mothers through pregnancy, birth, and postpartum. In 2021, Virginia adopted legislation to certify doulas to provide professional services to pregnant women through the first postpartum. The Virginia Certification Board certifies doulas who provide documentation demonstrating their completion of a mandatory training curriculum offered by approved providers (Virginia Department of Health, n.d.). An inquiry to the Virginia Department of Health indicates that health promotion and prevention knowledge areas cover general topics, but they do not specify direct training in safe sleep. The State does not require education on safe sleep practices (C. Staton, personal communication, February 14, 2023). The omission of safe sleep practices from training requirements may be a significant oversight in the certification program.

Black caregivers increasingly turn to doula services. Ogunwole et al. (2020) show that Black caregivers particularly seek doulas to address racial bias in medicine. Doulas in Black communities are often trusted members of the communities who provide culturally responsive care grounded in principles of social justice. They indicate doulas as promising resources to reduce the rates of Black infant mortality (Ogunwole et al., 2020).

## Review of the Literature

Our thorough review of the literature found a critical lack of research on doulas and their methods for putting infants down for sleep. This finding is consistent with other studies pointing to an overall paucity of literature on doula services (Falconi et al., 2022; Ogunwole et al., 2020). We found two articles that explicitly addressed the practices of doulas in putting infants down for rest.

One study surveyed almost 700 Chicago families to explore their compliance with the American Academy of Pediatrics (AAP) infant safe sleep guidelines (Jacobson, 2022). They found that parents are often involved in unsafe sleep practices with their infants. The authors highlighted distrust and miscommunication as the reasons why families reject safe sleep recommendations from medical providers. They concluded that alternative medical professionals such as doulas might have unique opportunities to explain to mothers and other family members the benefits of safe sleep practices and to offer mothers strategies for soothing infants who seem uncomfortable on their backs. While the Jacobson article does not explicitly focus on Black parents and has limited implications for the role of doulas, it provides some limited insight into parental attitudes towards safe sleep practices, which are relevant to the broader discussion on doulas and infant sleep practices.

A second study that arose from the literature review examined the impact of doula-home- visiting on birth outcomes, postpartum maternal and infant health, and newborn care practices in a randomized controlled study (Hans et al., 2018). Across a diverse sample of over 300 mothers, the researchers found that the doula-home-visiting intervention was associated with positive infant-care behaviors, including increased safe sleep practices (Hans et al., 2018). This study lends additional support for the use of doulas in promoting safe sleep.

The researchers found only two studies explicitly addressing doulas’ practices for putting infants down for sleep. Both support the use of doulas in promoting safe sleep, however the researcher drew these conclusions based on doulas specially trained in safe sleep. Particularly given the diversity of doulas and their backgrounds, additional information is needed to understand the training and practices of doulas in putting infants down for rest. This study intends to fill the gap in the literature. Medical professionals and other conventional helpers can likely learn from the practices of doulas in promoting safe sleep. It is also possible that doulas have areas in which they can continue to learn related to safe sleep.

## Study Purpose and Objectives

The purpose of this study was to explore doulas’ perspectives and practices in the field of putting infants down to sleep. The researchers aimed to determine whether Black caregivers that work with doulas are likely to encounter safe sleep education. The objectives were: (1) To explore how doulas are prepared to provide information about safe sleep practices, (2) To explore how doulas promote safe sleep recommendations with their clients, and (3) To identify recommendations for policies and practices.

The setting of this study was conducted primarily online. It used a descriptive approach. The design fit the line of investigation because of the lack of existing research on the topic. Descriptive research allows researchers to explore topics that are less understood or understudied. It helps in gaining initial insights and understanding of the phenomenon before delving into deeper investigations. Descriptive research emphasizes understanding a phenomenon within its natural context. This approach provides a rich backdrop that allows the researchers to interpret and analyze the data more meaningfully. By focusing on detailed descriptions, the researchers can uncover underlying patterns, trends, and relationships that might not be evident in quantitative research.

## Methodology

### Research Design Overview

The Institutional Review Board (IRB) at the researchers’ institution reviewed and approved this study. The research was conducted in accord with prevailing ethical principles and as approved by the Institutional Review Board. In addition, the researchers referred to the COREQ Checklist to ensure adherence to qualitative study criteria. While all standards were met, participant checking was omitted from the study protocol, which means participants were not provided the opportunity to offer feedback on the findings. This omission may warrant consideration in future research endeavors.

The researchers conducted three focus group meetings. The focus group meetings were 60 min long and took place virtually through Zoom. Focus group questions were semi-structured. Questions asked included “How do you recommend that caregivers put infants down for sleep?” and “What is your understanding of the safe sleep recommendations from the American Academy of Pediatrics (AAP)?” See appendix A for the interview guide.

### Researcher Description

Two researchers, one primary researcher and a research assistant, conducted this study. Both identify as Black women. The primary investigator is a licensed clinical social worker and a professor for an institute of higher education. She has prior experience planning and facilitating focus groups. While she is professionally trained as a social worker, she has a critical understanding of the cultural distrust of helping institutions and recognizes the value of alternative medicine to include doulas in Black communities. She practices alternative medicine in conjunction with conventional treatments. She is familiar with ASSB from her professional experience. She had no experience with doulas prior to this study.

As a member of a regional Child Fatality Team Review, she observed an emerging trend in infant deaths. She began to note documentation describing mothers of Black SIDS victims as non-compliant with conventional healthcare providers or having low service utilization. Being familiar with the trend toward alternative medicine among Black people who are more distrusting of conventional medical providers, the primary investigator questioned whether the mothers were working with doulas. Unfortunately, the Child Death Review Case Reporting Form does not collect information to inform this question. She further questioned whether the mothers under the care of doulas would routinely receive information about safe sleep from their providers. This study emerged from this particular line of inquiry, which was made more significant in light of legislation in Virginia to include doulas’ services in Medicaid coverage.

The research assistant is an undergraduate student majoring in social work. She was chosen by the primary researcher for her exemplary academic performance and competence in the field. This project served as her first experience leading a focus group, though she had previously engaged in small group facilitation as part of her coursework in social work. The primary researcher offered her training and guidance to facilitate the focus groups.

The research assistant had no prior experience with doulas. The research assistant learned about ASSB from textbooks and lectures in college. Before college, she was familiar with generational misconceptions about safe sleep practices. Having been presented with new information about safe sleep recommendations from the AAP, she is committed to promoting best practices for putting infants down for sleep, particularly in Black communities. She recognizes the need to counter generational knowledge antagonistic toward safe sleep practices, however she is also aware of the cultural distrust of medical professionals in Black communities. Having been introduced to doulas through her studies, the research assistant believes doulas may be instrumental in helping to advance safe sleep practices in Black communities because they are trusted professionals. As such, she is driven to learn more about how doulas practice and where there may be gaps and opportunities to expand their services. This project benefits from her curiosity, particularly in data analysis.

### Researcher-Participant Relationship

The researchers recruited some participants from their personal and professional networks. The researchers did not discuss the topic with the participants before the focus group or share the questions with them before the meeting. During recruitment, the researchers advised that the focus group would explore their training and practices in the field. The researchers compensated participants for their time and effort with a $25 Visa gift card.

### Participant Recruitment

This study used purposive and snowball sampling to recruit focus group participants. The recruitment process was electronic. The researchers sent an initial invitation to their personal and professional networks. The researchers invited them to share the invitation with doulas who fit the eligibility criteria. With those who reached out to the researcher, the consent form served as a guide for the discussion. Recruitment was a back-and-forth conversation between a member of the research team and the prospective subject, involving providing information to the individual and then questions and answers. The researchers explained to prospective participants the purpose of the focus group, the eligibility criteria, the risks and benefits, and contact information. The process fundamentally did not adhere to a fixed script. The researchers recruited 10 focus group participants for the first meeting, 11 for the second, and 11 for the third, totaling 32 respondents. Each participant gave informed consent to participate in the study.

### Participant Selection

Because this study was particularly interested in understanding Black populations who may distrust conventional medicine, the researchers limited participation to (1) Doulas who self-identify as Black and (2) Doulas who work predominately with Black expectant or new mothers. To align with the research and the observations that informed this study, the researchers excluded those who practice outside these parameters.

### Sample

No demographic data was collected from the focus group participants. The first focus group included doulas at various stages of certification from non-state-certified to state-certified. Participants in the second focus group were predominantly certified doulas practicing in Virginia. The final sample size for the first focus group was six, five for the second, and six for the third group. Together, the total sample size of participants was 17. The retention rate was 53%. Three invited participants reported that they could not attend the meeting due to unexpected occurrences in the field. One noted that she was not able to participate because of technical difficulties. Some participants noted that they were acquainted with each other, while others mentioned that they were meeting for the first time. One of the participants was a relative of the primary researcher. None of the participants had prior knowledge of the study or its questions before participating.

### Data Collection and Analysis

Because the focus groups were held virtually, the researchers carefully monitored the interactions, level of engagement, and non-verbal-cues of the participants to ensure good group dynamics. The focus groups were audio-recorded and transcribed. The researchers halted data collection when they achieved saturation. The researchers assessed saturation as the point in which issues begin to be repeated and further data collection becomes redundant (Hennink et al., 2019). The researchers cleaned and de-identified the transcripts as needed.

The researchers utilized both Artificial Intelligence (AI)-driven coding tools and manual coding techniques. The transcriptions and observation notes from each focus group were independently reviewed and coded by the two researchers. This process ensured that insights were not influenced by the perspective of a single coder, reducing the potential for bias.

The researchers used the Artificial Intelligence-driven tools available through ATLAS.ti Web to automatically perform open and descriptive coding for each focus group transcript. After AI coding was completed, the generated codes were compared with those identified through manual coding. This comparison aimed to identify areas of agreement and disagreement between the two coding approaches. The researchers resolved disagreements through discussion and the creation of additional codes, ensuring that all relevant themes and patterns were captured. The final step involved organizing the codes into thematic categories. This process involved synthesizing similar codes into broader themes, providing a structured framework for analyzing and interpreting the data. By triangulating the data through these methods, the researchers were able to validate the findings and ensure the reliability and trustworthiness of the study results. This approach allowed for a comprehensive examination of the data from multiple perspectives.

## Findings

The analysis resulted in the development of four themes related to infant safe sleep: (1) Individualized Services, (2) Cultural Sensitivity, (3) Negotiating Safety, and (4) Safe Sleep Education. No subthemes were identified. Response patterns remained consistent across participants, with no divergent responses noted.

### Individualized Services

A top theme that connected various codes was individualized services. This theme focuses on the strategies and practices employed by doulas to tailor their support to the unique needs and circumstances of each client. Content relevant to this theme included discussions surrounding the customization of care plans, the importance of home visits for comprehensive assessments, and examples of how doulas adapt their approach based on their understanding of clients’ preferences and challenges. Participants also related that they follow caregivers and their infants and get to know them in a way that involves detailed knowledge. One focus group participant described the importance of home visits, saying,

The home visits are so important, I know sometimes doulas like to try and do zoom or virtual visits, but it’s so important to get in the household to see [and] make sure, you know, prior to delivery, that the space is set up for baby and that Mom has everything they need, so that after delivery [the] baby can be safe.

As a result of their intimate knowledge, doulas can tailor their services to fit the particular interests of their clients. The participant expressed that conducting visits in the home offers a unique perspective into the family’s environment, where they are able to assess for risk and protective factors. She went on to provide the example: if she knows there is a smoker in the home or smells evidence of smoke, she can encourage them not to co-sleep with their infant or expose them to second-hand smoke because it may weaken their respiratory system and put them at greater risk for SIDS. The doulas believed working with caregivers in their homes also facilitates greater trust and openness from the caregiver. Doulas remarked on their tendency to elicit more honest and transparent information from their clients, allowing them to direct their clients better, saying,

There is less of a performance, like people get to be real about their practices at home. People get to tell you: ‘You know this is what I like to do. I know the doctor said this, or I know you know the research said this, but this is what I’m doing.’ It’s kind of a better ability to meet people where they want to be in their family and their preferences.

Because of their trusted position, doulas described caregivers as more receptive to the information and education that doulas provide. They also advised that they offer caregivers instrumental support. A study participant mentioned, “My role and my experience have been a lot of listening, a lot of affirming, a lot of questions, asking questions, answering, and offering support in that way. And just being present.”

### Cultural Sensitivity

Cultural sensitivity emerged as a prominent aspect of doula practice, encompassing doulas’ awareness, respect, and responsiveness to the cultural backgrounds and beliefs of their clients. Relevant content for this theme included discussions on how doulas navigate cultural differences, collaborate with extended family members, and advocate for culturally appropriate care within healthcare settings. Doulas described themselves as being aware of and accepting of their clients’ cultural values and beliefs. One participant said “I’m always the doula, first of all, but the way that we interact, I’m a mother figure, a homegirl that we go walking to help calm the baby down and keep the mom in shape. So, I’m a motivator.” Likewise, a participant mentioned that “there are fewer touch points for those medical providers; you kind of have a little more access.” They suggested that their cultural sensitivity helped their clients to feel more comfortable and familiar with them, which they believed translated to greater compliance with their professional recommendations: “I’ve got a good feel for the family, so I might be able to say something like you know you can’t hold that baby like that in the car.” Participants also noted that they collaborate with the larger family system and help to advise extended family on caring for the infant. One participant said: “We have to educate the grandparents, the parents, the partner so that they can understand incorporating the whole family and educating all the support systems so that they can be aware of the reason why.”

Regarding cultural sensitivity, doulas emphasized the importance of addressing historical trauma and providing advocacy. They indicated that their clients were wary and distrusting of medical providers because of a history of medical bias and mistreatment in Black communities. For example, one participant related: “The women of color are always like, ‘I hear the statistics and I hear the stories, the horror stories and I just want to have someone there to make sure that I’m safe.’” To combat these concerns, participants appreciated the importance of advocating for their clients. One shared:

I had a client who the doctors were pushing for her to have a C-section, and that’s not what she wanted. So I actually pulled out her birth plan and put a doctor to the side and said, ‘That’s not what my client is asking for.’ I didn’t want to speak too much, as if I was the client, so I spoke to my client to let her know that you know this is your birth. You have to, you know, speak up for yourself. So we spoke to the doctor together and he came out fine.

### Negotiating Safety

One of the key findings from this study was how doulas negotiate safe sleep with their clients. This theme explores doulas’ approaches to facilitating discussions and decision-making around safe sleep practices with their clients. Content relevant to this theme includes descriptions of how doulas address caregivers’ concerns, provide education on safe sleep guidelines, and collaborate with clients to implement practical strategies for promoting infant safety. Overwhelmingly, participants indicated a desire to promote safe sleep and reinforce AAP recommendations in the field. For example, one stated: “I usually tell them what is recommended. Some things that are non-negotiable.” One participant remarked that some caregivers internalize significant guilt and anxiety about not upholding safe sleep recommendations:

They do a very long thing about it in the hospital before you leave. They make sure you know how the baby is supposed to go to sleep. That’s why I know that there is a lot of guilt that comes when it does not go that way. When she gets home.

Another participant added: “Then there comes this guilt. When you’re feeding your baby, fall asleep with your baby next to you, and then it’s like the parents are all stressed out.” In contrast to feelings of guilt, participants pointed to a critical conflict between medical advice and the practices of caregivers. They described strategies for helping caregivers arrange for safe sleep through discussion and problem solving. In particular, one participant pointed to small environmental changes to promote safe sleep:

“I try to start smaller. So maybe it’s just like the outfit that they’re going to sleep in. Maybe it’s uncomfortable. Maybe there’s a tag sticking somewhere. Maybe if the baby is swaddled, they don’t like the swaddle. Maybe we can adjust the temperature. I try to do environmental things first. Just maybe the baby, may like a sound machine. Maybe they don’t like the frequency of the sound machine. Just small things before changing things like, you know, baby sleeping on back and sleeping on a harder surface. Maybe the lighting is wrong, like just small. Maybe they need to be burped two or three times before they’re laid down on their back. That one burp wasn’t enough. So, I try to have them tweak the smaller things first before going to the big, you know, changing their room. They’re sleeping in the position. They’re sleeping in things like that.”

Participants also discussed their methods for minimizing risk in sleep environments. One shared:

I learned about pacifiers being a useful thing to help prevent things like SIDS. But later on down the line, if, like the parent is breastfeeding, they are supposed to wait at least 8 weeks before. And 2 weeks before introducing a pacifier.

They also related that they monitor the use of nursing pillows and other infant products to ensure compliance and safety. In addition to minimizing risk, doulas also promoted protective factors. Doulas recognized the value of breastfeeding and skin-to-skin contact in promoting infant safety. They related providing information on proper breastfeeding techniques and positions that ensure safety during sleep. In addition, they shared that they discuss safer options for co-sleeping such as room-sharing and using safe co-sleepers.

### Safe Sleep Education

Safe sleep education encompasses doulas’ knowledge acquisition processes and sources of information related to infant safe sleep practices. Relevant content for this theme included discussions on formal training experiences, experiential learning through client interactions, peer networking for knowledge sharing, and independent research efforts to stay updated on evidence-based practices. Some doulas indicated that they knew about safe sleep through formal training in the process of becoming state-certified; however, they did not specify a trainer or provider. A couple pointed to a community provider that was instrumental in helping them to navigate the training and certification process. Others reiterated the conflict between best practices and the practices of caregivers in the field. One participant described learning through experience to help caregivers find ways to make safe sleep more accommodating to their needs and lifestyle.

When I was trained, I was trained like no co-sleeping, not an option, no way to go around it. But then, after experience and talking to more experienced midwives. I just learned more about safe co-sleeping and how to make it an option, because, after working, you know, with a couple of families, you just see how sleep deprived the parents get because you, and especially in the beginning, when babies are really grunty, and they’re just kind of moving around. It will wake you up at the smallest sound, especially being on the monitor and not having them close, whereas when they’re close, you can get used to the grunts and the moving and stuff like that. So yeah, a mixture of training and experience has kind of molded my thoughts on co-sleeping.

Some participants indicated a peer network and learning from other practitioners. For example, one said:

I think that it is important to continuously learn and look for resources. Even touching base with the midwives that I’m connected with on what works, because everybody is going to do something different.

Another participant shared they learned through “watching my mentors talking to my [clients] kind of listening to their conversations and, you know, how they talk with their patients and things like that.” One indicted that “It’s very important to be connected with other doulas, because that’s how we learn.” “I also look at the evidence based Youtube videos and her podcast and then also word of mouth. If I hear other doulas talking about a specific practice, or, you know, suggestion, I’ll look it up for myself just to get more information so that I can possibly share that with the mom that I work with.” Others described researching the topic independently. They read professional pamphlets, brochures, and research reports. One participant noted: “I am consistently researching. I am on evidence-based birth practices. I am looking up scholarly articles and research to continue to educate myself.” Another reported: “I remember, like going through all these different health communication campaigns of like, you know, cry it out or back to sleep campaigns and just really studying those pamphlets to understand what kind of language was being communicated in them.”

## Discussion

Doulas are trusted practitioners that Black caregivers rely on for support, knowledge, and advocacy. As such, they are instrumental in supporting hard-to-reach communities that are more distrusting of conventional health providers. Doulas play a crucial role in providing support and education to expectant and new caregivers, including guidance on safe sleep practices for infants, as established by the AAP. Doulas emphasize the importance of minimizing risk and promoting the safety of infants in a sleep environment. Doulas listen to the caregivers’ concerns and answer any questions related to safe sleep practices. They help parents make informed decisions that prioritize their infant’s safety. Doulas stress the importance of consistent safe sleep practices both at home, during travel, and with extended family. Doulas respect and acknowledge the cultural practices and beliefs of the family they are supporting. They work collaboratively with caregivers to find safe sleep solutions that align with cultural and individual preferences while maintaining a focus on infant safety.

### Negotiating Safe Sleep

This study found that doulas play a critical role in negotiating safe sleep. Doulas assess risk and protective factors and evaluate them against the environmental conditions and help caregivers implement the most practical strategies. The practice of negotiating safety is particularly important for times in which the family experiences disruption or chaos, which may raise the risk for ASSB. This finding supports the move away from prohibitory language against co-sleeping, in particular (McKenna & McDade, 2005). Previous studies indicate that recommendations such as absolutely no bed-sharing is misleading and belies intuition, generational knowledge, and anecdotal evidence (McKenna & McDade, 2005). Unqualified recommendations are more damaging than helpful. Doulas help caregivers understand the best practices and incorporate them into their practices. The process of negotiating sleep fundamentally does not include a firm and hard rule. It is a mutual discussion and arrangement of the terms of putting the infant down for sleep. Doulas should give consideration to small environmental changes that may be made to make sleep recommendations more practical. They should continuously revisit and revise the sleeping arrangements with caregivers to fit the evolving needs of the family system.

### Training Recommendations

The doulas demonstrated a commitment to promoting safe sleep. They related consistently and regularly reinforcing safe sleep. They appeared knowledgeable about the importance of safe sleep as well as safety and risk factors. While some indicated that they learned about safe sleep from formal trainings, more pointed to experiential learning, mentorship, and self-study. This finding indicates a need for continuous education on safe sleep. To support doulas in their efforts in this area, more formal training on safe sleep is warranted. Importantly, the participants pointed to a discrepancy between the best practices and the desires and practices of caregivers in the field. Participants indicated that they resolved this conflict through experiential learning. Working with families, they identified changes required to their skills, attitudes, and practices to meet the needs of the families they serve. Given the urgency of safe sleep, training should support doulas in adjusting their practices to meet the needs of families while supporting safe sleep. Training should focus on individualizing services, listening to and collaborating with families, and negotiating safe sleep. Doulas should be trained to bring about safe sleep through discussion with caregivers. They should receive education on the safety and risk factors and ways to problem solve around safe sleep.

### Recommendations for Future Studies

Conclusions drawn from this study should be taken with caution given its small sample size. For further insight, a more robust study is recommended. Aside from using a larger sample size, future studies should explore the quantity and quality of safe sleep content in available trainings. In addition, there is merit in examining other practices, such as nutrition and health. Doulas serve a critical role in the lives of new mothers and infants. Additional research is warranted to explore if and how to doulas influence various aspects of their clients’ lives.

### Limitations

This study has a number of limitations that should be considered. This study used purposive and snowball sampling techniques that may lead to a biased sample, as participants are selected based on specific criteria and through referrals from existing participants. This could result in overrepresentation or underrepresentation of certain perspectives within the focus groups. In addition, participants who voluntarily respond to the invitations or referrals may have unique characteristics or experiences compared to those who do not participate, leading to self-selection bias and potentially skewing the findings. The final sample size may be considered relatively small. This could limit the generalizability of the findings and the ability to capture a diverse range of perspectives within the target population. It should also be noted that all participants identified as Virginia-based practitioners. This further limits the generalizability of the study findings. Conducting the focus groups online introduced the possibility of technical issues such as poor internet connectivity, audio/video disruptions, and difficulties navigating the virtual platform. One participant, for instance, reported being unable to join due to technical difficulties on her end.

### Strengths

This study has a number of strengths that should be noted. Though the final sample was relatively small, the study met saturation. Recruiting participants for multiple focus group sessions enhanced the richness and depth of the data collected, allowing for a more comprehensive exploration of the research topic. Along those same lines, having the focus groups online increased accessibility for participants who may have mobility limitations, live further from the researchers, or have scheduling constraints. The researchers also specified participants who met specific criteria relevant to the research focus. By targeting doulas involved in maternal care, the study ensured that participants had relevant expertise and experiences related to the study topic of infant safe sleep practices. Moreover, by studying practitioners in Virginia, the researchers can shed light on the implications for Virginia policies, particularly in light of their recent legislation in this area. This research can provide valuable insights to inform the implementation and potential expansion of doula services in Virginia.

## Conclusion

This study conducted three focus groups to explore the practices and training related to safe sleep among doulas in Black communities. This study identified four themes: (1) Individualized Services, (2) Cultural Sensitivity, (3) Negotiating Safety, and (4) Safe Sleep Education. These themes have essential implications for practices and policies. Doulas should promote safe sleep and be prepared to negotiate it with their clients. For them to be effective in this role, they have to be well-trained. The state should make training freely available for doulas at various levels of practice. It should emphasize best practices while teaching them how to problem solve and identify environmental solutions for safe sleep. Training supports the vital role that doulas play in Black communities.

### Electronic Supplementary Material

Below is the link to the electronic supplementary material.

Supplementary Material 1

## Data Availability

The data generated during the current study are not publicly available but are available from the corresponding author on reasonable request.
