# Supplementary material for: Promoting Safe Sleep: The Role of Doulas in Black Communities
Source: Matern Child Health J. 2024 Jun 4;28(8):1422–31. doi: 10.1007/s10995-024-03928-0 (PMC11269406; doi:10.1007/s10995-024-03928-0)
Supplement: Supplementary file 1 — Supplementary Material 1 [file 10995_2024_3928_MOESM1_ESM.docx]

**Appendix A: Interview Guide**

**Introduction**

- Welcome and thank participants for joining.
- Briefly introduce the purpose of the focus group: to gather insights on infant sleep practices and the role of doulas in promoting safe sleep.
- Emphasize that all perspectives are valued, and participants should feel free to share openly.
- Read and have participants sign the informed consent form. Ensure participants understand their rights and the purpose of the study before proceeding.
- Inform participants that they will be compensated with a Visa gift card at the end of the session via email.

**Questions**

1. What makes expectant mothers want to work with you.
   1. Probe: What type of services do you provide?
   2. Probe: How are your services culturally sensitive?
   3. Probe: What is the cultural leaning of your services?
   4. Probe: How long have you been providing services?
2. What is your experience with putting infants down for rest?
   1. Are mothers typically receptive of safe sleep recommendations?
   2. Are babies typically agreeable to sleeping independently and on their back?
3. How do you recommend that caregivers put infants down for sleep?
   1. Probe: What influences your decision-making?
   2. Probe: What are some difficulties mothers have putting babies to sleep?
   3. Probe: How do you advise that mothers address those challenges?
   4. Probe: What are some reasons you would support or recommend co-sleeping or other alternatives to safe sleeping practices?
   5. Probe: Under what circumstances do you recommend co-sleeping or other alternatives to safe sleeping practices?
   6. Probe: What strategies do you promote for soothing babies who seem uncomfortable on their backs?
   7. What do you do when your recommendations conflict with what elders or family members suggest?
4. What is your understanding of the safe sleep recommendations from the American Academy of Pediatrics (AAP)?
   1. Probe: Do you agree with the safe sleep practices?
   2. Probe: Have you received formal education or training around safe sleep practices? How did you learn about safe sleep practices? What do you know about safe sleep practices?
   3. Probe: Where did you learn about safe sleep practices?
   4. Probe: How do you advise a parent when a baby is fussy or resistant to sleeping independently or on their back?
   5. Probe: What strategies do the mothers under your care typically practice for putting their baby down for sleep?
   6. Probe: What do you do when your recommendations conflict with what elders or family members suggest?
   7. Probe: What are some factors that you consider before allowing or endorsing a parent to co- sleep with their child?
   8. Probe: What are some red flags or indicators that a parent is cosleeping dangerously?
   9. Probe: What helps parent to practice safe sleep?
   10. Probe: What prevents mothers from practicing safe sleep?
5. What do you look for in regards to bedding or a sleeper for babies?
   1. Probe: What concerns do you have about infant products or their use?
   2. Probe: How do you monitor their use?

**Closing**

- Thank participants for their time and valuable contributions.
- Provide contact information for any follow-up questions or concerns.
- Review and sign the informed consent form again to confirm completion of the interview process.
